# Regional lymph node changes on breast MRI in patients with early-stage breast cancer receiving neoadjuvant chemo-immunotherapy

**DOI:** 10.1007/s10549-024-07481-w

**Published:** 2024-09-21

**Authors:** Saya Jacob, Anika Christofferson, Samantha Fisch, Peter Norwood, Paolo Castillo, Hongmei Yu, Gillian Hirst, Hatem Soliman, Rita Nanda, Rita A. Mukhtar, Cheryl Ewing, Melanie Majure, Michelle Melisko, Hope S. Rugo, Laura Esserman, Elissa Price, A. Jo Chien

**Affiliations:** 1 University of California San Francisco Comprehensive Cancer Center, 1825 4 Street, San Francisco, CA 94158 USA; 2 University of California San Diego, 9500 Gilman Dr, La Jolla, CA 92093 USA; 3 Quantum Leap Healthcare Collaborative, 499 Illinois Ave, Suite 200, San Francisco, CA 94158 USA; 4 Moffit Cancer Center, 10920 N. McKinley Drive, Tampa, FL 33612 USA; 5 University of Chicago, 5841 S. Maryland Avenue, Chicago, IL 60637 USA

**Keywords:** Immunotherapy, Breast MRI, Early-stage breast cancer, neoadjuvant chemotherapy

## Abstract

**Purpose:**

Establishing breast MRI imaging patterns associated with neoadjuvant immunotherapy is needed to monitor response. We analyzed serial breast MRIs in patients receiving neoadjuvant chemo-immunotherapy on the I-SPY2 clinical trial.

**Methods:**

Patients with stage 2–3 HER2-negative breast cancer were randomized to weekly paclitaxel (control), weekly paclitaxel and pembrolizumab, or weekly paclitaxel, pembrolizumab and intra-tumoral injection of SD-101, a TLR9 agonist. All patients received AC. Regional lymph nodes were retrospectively evaluated on breast MRI at baseline, 3, 12 and 20 weeks by a single blinded radiologist. MRIs were assessed for development of new regional lymphadenopathy, or increase in the longest diameter or cortical thickness of the largest abnormal regional lymph node.

**Results:**

Between 12/2015 and 4/2021, a total of 43 patients enrolled in the control (*n* = 16) and paclitaxel + pembrolizumab ± SD-101 (*n* = 27) arms. 12 of 27 patients (44.4%) receiving chemo-immunotherapy experienced increased lymphadenopathy within the first 12 weeks compared to 1 of 16 patients (6.3%) in the control group (*p* = 0.014). Most patients with increased lymphadenopathy were in the SD101/pembro arm (*n* = 10, *p* = 0.002). Increased lymphadenopathy was observed despite concomitant decrease in breast tumor size at all time points. 11 of 12 patients with increased lymphadenopathy had pathologically negative nodes at surgery. There was no association between lymphadenopathy and lower residual cancer burden or immune-related toxicity.

**Conclusions:**

The combination of neoadjuvant paclitaxel and pembrolizumab ± SD101 intratumoral injection was associated with early increases in regional lymphadenopathy on MRI despite decreased breast tumor size. Increased lymphadenopathy was not associated with node positive disease at surgery.

**Supplementary Information:**

The online version contains supplementary material available at 10.1007/s10549-024-07481-w.

## Introduction

The addition of immune checkpoint inhibitors (ICI) to chemotherapy has improved overall survival for patients with metastatic triple negative breast cancer (TNBC), 5-year event-free survival in early-stage TNBC, and improved pathologic complete response rates in high-risk hormone receptor positive (HR +) disease [1–4]. These agents work by blocking the immunosuppressive interaction between the programmed death 1 (PD1) receptor on effector T cells and the programmed death ligand 1 (PDL1) within the tumor microenvironment thereby promoting anti-tumor immunity and tumor cell death [5]. However, despite these advances, immune-related adverse events (irAEs) can occur in up to 50% of patients and serious, irreversible side effects have been reported including adrenal insufficiency, insulin-dependent diabetes and neurologic events [6, 7]. In addition, not all patients require immunotherapy plus chemotherapy to achieve excellent outcomes. For example, up to 51% of patients with stage II–III triple negative breast cancer (TNBC) who were treated with chemotherapy alone achieved pathologic complete response (pCR) at the time of surgery, and of these patients, 92.6% were event-free at 3 years [1]. Thus, early markers of response to immunotherapy are needed in order to identify patients who do not benefit and who can be spared from unnecessary immune toxicity.

In the neoadjuvant setting, serial imaging has evolved as an important tool to assess response to treatment and may be one potential marker of early response [8]. Of the available standard imaging methods, MRI has been demonstrated to have higher sensitivity for tumor extent and response compared to mammogram, ultrasound or clinical exam with an estimated sensitivity for detecting pathologic complete response of 0.88 (95% confidence interval {CI}, 0.78–0.94) [9–15]. Quantitative functional tumor volume (FTV), defined as the volume of tumor displaying rapid early enhancement followed by a rapid loss of enhancement on dynamic contrast-enhanced MRI, represents an additional novel radiologic marker of response and is assumed to represent the area of viable tumor cells [16]. Changes to FTV have previously been linked to response to neoadjuvant therapy [16–19].

The Investigation of Serial Studies to Predict Your Therapeutic Response With Imaging And moLecular Analysis 2 (ISPY2) phase II clinical trial aims to identify effective novel agents using personalized assessment of tumor molecular features for patients with high-risk breast cancer in the neoadjuvant setting [20]. Patients enrolled in the trial are randomized based on their tumor molecular subtype to receiving one of several investigational agents in combination with paclitaxel for 12 weeks followed by four cycles of dose dense doxorubicin/cyclophosphamide (AC) before proceeding to surgery. While on-treatment, patients undergo serial breast MRIs to assess treatment response. Among the investigational agents used were the PD-1 monoclonal antibody pembrolizumab, as well as intra-tumoral injections of SD-101. SD-101 is a therapeutic vaccine that contains a synthetic oligonucleotide with cytidine-phospho-guanosine (CpG) motifs that stimulates plasmacytoid dendritic cells (pDC) through engagement of toll-like receptor 9 (TLR9) [21]. Preclinical studies have demonstrated that activation of plasmacytoid dendritic cells expressing TLR9 by SD-101 potentiates T cell infiltration into the tumor microenvironment, thereby increasing tumor cell destruction [22, 23]. Prior early phase clinical trials evaluated SD-101 in combination with pembrolizumab in patients with melanoma and lymphoma demonstrating response rates ranging from 28 to 78% with a favorable safety profile [24, 25].

While imaging changes after immunotherapy have been described in other tumor types [26], there are little data to describe changes to imaging in patients with early-stage breast cancer. This is particularly true of the changes seen with regional lymph nodes in response to treatment. An understanding of these changes is critical to guiding response assessment and planning surgical treatment in patients undergoing neoadjuvant chemo-immunotherapy. In particular, an understanding of the significance of emerging lymphadenopathy with this therapy and whether this represents progression vs inflammatory changes. In this study, we aimed to describe regional lymph node changes on MRI in patients with Stage 2–3 breast cancer receiving standard chemotherapy with or without immunotherapy on the I-SPY2 trial. We hypothesized that patients undergoing treatment with neoadjuvant chemo-immunotherapy would demonstrate increased regional lymphadenopathy, possibly due to underlying immune activation.

## Methods

### Participants and study design

#### I-SPY2 trial

The I-SPY2 trial is a multi-center neoadjuvant platform trial testing novel agents in combination with chemotherapy in patients with Stage 2–3 biopsy-proven breast cancer. The I-SPY2 trial design has been reported previously [27, 28]. I-SPY2 enrolls patients with all receptor subtypes. Participants were 18 years of age or older, had an Eastern Cooperative Oncology Group (ECOG) performance status of 1–2, adequate end-organ function. The study protocol was reviewed and approved by the institutional review board (**UCSF IRB# 23-40,166; NCT# 01042379)** and all patients provided written informed consent prior to study enrollment.

Patients on the I-SPY trial are randomized based on their tumor molecular characteristics to either the control arm consisting of standard chemotherapy with weekly paclitaxel 80 mg/m^2^ for 12 weeks followed by 4 cycles of doxorubicin 60 mg/m^2^ and cyclophosphamide 600 mg/m^2^ (ddAC) every 2 weeks for 8 weeks prior to undergoing definitive breast surgery, or an investigational arm. Investigational arms consist of weekly paclitaxel in combination with one of several different investigational agents for a total of 12 weeks followed by 4 cycles of AC and surgery.

#### Lymph node assessment sub-study

This study included patients at a single institution (University of California San Francisco {UCSF}) randomized between 12/2015 and 4/2021 to either: (i) the control arm; (ii) investigational pembrolizumab 200 mg IV every 3 weeks combined with weekly paclitaxel; or (iii) investigational intra-tumoral injection of SD-101 given weeks 1–4, 7, 10, in combination with weekly paclitaxel and pembrolizumab 200 mg IV every 3 weeks. The dose of SD-101 (2mg/ml) was determined by the tumor size (1 ml for tumors < 5 cm, 2 ml for tumors ≥ 5 cm or with direct extension into chest wall/skin).

### Imaging assessments

As part of I-SPY2, patients underwent dynamic contrast-enhanced breast MRI at baseline (pre-treatment), as well as 3, 12, and 20 weeks on study therapy. The 20-week scan served as the patient’s pre-operative evaluation. For this investigation, each MRI was reviewed by a single UCSF breast radiologist who was blinded to the treatment arm as well as the follow-up MRI time point after the baseline scan. MRIs were evaluated for abnormal ipsilateral axillary nodes, defined as nodes with cortical thickening > 3mm, effacement or loss of the fatty hilum, rounded shape, and irregular margins. MRIs were monitored over time for changes in size of the largest abnormal node OR the development of new abnormal nodes. New lymphadenopathy was defined as development of new qualitative morphologic abnormalities as assessed qualitatively by the reading radiologist. Morphologic abnormalities included nodal enlargement due to circumferential or eccentric cortical thickening > 3mm, effacement or loss of fatty hilum, rounded shape, nodal soft tissue replacement. Evaluation of new lymph nodes included axillary levels level I–III. Longest tumor diameter of breast tumor was also assessed at each time point by the same radiologist.

FTV of the primary breast tumor was assessed centrally prospectively during the trial and reported for each timepoint. FTV was defined as the volume of tumor displaying rapid early enhancement followed by a rapid loss of enhancement on dynamic contrast-enhanced MRI [16–19].

### Clinical and pathologic assessments

Several clinical and pathologic characteristics were collected at baseline. These included residual cancer burden (RCB) at time of surgery [29], estrogen receptor status, clinical node status pathologic grade of breast tumor at diagnosis, and MammaPrint [30] high-risk category (with “high 1 [H1]” indicating patients with a score of 0 to − 0.57 and “high 2 [H2]” indicating patients with a score less than − 0.57). Tumor specimens were also analyzed using the ImPrint immune assay, an investigational 53 gene signature developed through the ISPY2 clinical trial aimed at identifying tumors most likely to achieve pathologic complete response with the addition of ICI [31, 32]. Incidence of immune-related adverse events was evaluated for patients based on regional lymph node change.

### Study objectives

The primary study objective was to characterize changes in regional lymph nodes on breast MRI and to determine if patients receiving chemo-immunotherapy had a higher incidence of new or enlarging regional lymphadenopathy during treatment compared to patients receiving chemotherapy alone. A key secondary objective was to describe changes to breast tumor size and volume with chemo-immunotherapy. Exploratory objectives included: (i) assessing the correlation of increasing regional lymphadenopathy with residual cancer burden (RCB) [29] at surgery; (ii) assessing the correlation between increasing regional lymphadenopathy and development of immune-related toxicities; (iii) comparing changes in regional lymphadenopathy with changes in longest tumor diameter and FTV of the primary breast tumor; (iv) assessing the correlation between increasing regional lymphadenopathy and clinicopathologic characteristics.

### Statistical methods

The association between increased lymphadenopathy and type of treatment received was tested using Fisher's exact test using R where the null hypothesis was that there was no relationship between increasing lymphadenopathy and treatment received. This was tested against the alternative hypothesis that increasing lymphadenopathy was associated with receiving chemo-immunotherapy. In addition, the association between increasing lymphadenopathy and the extent of RCB was also tested using Fisher's exact test using R where the null hypothesis was that there was no relationship between increasing lymphadenopathy and achieving RCB 0 or 1. This was tested against the alternative hypothesis that increasing lymphadenopathy was associated with achieving RCB 0 or 1. A significance level of 0.05 was set for these tests. Descriptive statistics were used to evaluate changes with regional lymph nodes in relation to incidence of immune toxicity, changes in tumor size, FTV and clinicopathologic characteristics.

## Results

### Patient characteristics

Between December 2015 and April 2021, a total of 43 I-SPY2 patients were enrolled to the control (*n* = 16), pembrolizumab (*n* = 11) and pembrolizumab + SD-101 arms (*n* = 16) at UCSF. Patient characteristics are outlined in Table 1. The median age across the entire population was 45 years, and 35 patients (81.3%) were pre/peri menopausal while 8 (18.6%) were post-menopausal. 25 patients (55.5%) had estrogen receptor positive (ER +) and human epidermal growth factor receptor 2 (HER2−) disease while 18 patients (40%) had TNBC. The majority of patients were non-Hispanic white (30 patients, 69.7%).

**Table 1:** Patient characteristics

	All (*n* = 43)	Control (*n* = 16)	Pembrolizumab^a^ (*n* = 11)	SD-101^b^ (*n* = 16)
Median age	45	42	48	46
Menopausal status				
Pre/Perimenopausal	35 (81.3%)	13 (81.2%)	9 (81.8%)	13 (81.2%)
Postmenopausal	8 (18.6%)	3(18.8%)	2 (18.2%)	3 (18.8%)
Tumor characteristics				
ER + /HER2−	25 (55.5%)	8 (50%)	7 (63.6%)	10 (62.5%)
TNBC	18 (40.0%)	8 (50%)	4 (36.4%)	6 (37.5%)
Race				
Non-hispanic white	30 (69.7%)	11 (68.8%)	8 (72.7%)	11 (68.6%)
Hispanic	6 (14.0%)	2 (12.5%)	2 (18.2%)	2 (12.5%)
Asian	5 (11.6%)	3 (18.8%)	0	2 (12.5%)
Other	2 (4.7%)	0	1^*^ (9.1%)	1^**^ (6.3%)
Clinical node status				
Positive	26 (60.5%)	8 (50%)	8 (72.7%)	10 (62.5%)
Negative	17 (39.5%)	8 (50%)	3 (27.3%)	6 (37.5%)
Mean baseline tumor largest diameter in cm (range)	4.78 (1.3–12)	4.66 (2–12)	5.09 (2.7–8.7)	4.69 (1.3–8)
Mean baseline functional tumor volume in cc (range)	21.36 (0.03–71.03)	17.48 (1.31–64.33)	24.37 (2.35–67.98)	23.19 (0.03–71.03)

^a^Pembrolizumab group received pembrolizumab combined with paclitaxel

^b^SD-101 received SD-101 combined with pembrolizumab and paclitaxel

*One patient in the pembrolizumab group identified as American Indian or Alaskan Native, **One patient in the SD-101 group identified as Black or African American

*ER* + estrogen receptor positive; *HER2 − *negative for HER2 amplification; *TNBC* triple negative breast cancer

Eight patients (50%) in the control arm, 8 patients (72.7%) in the pembrolizumab group and 10 patients (62.5%) in the SD-101 arm were clinically node positive. Clinically node positive disease was confirmed by FNA. Mean baseline tumor diameter in the control, pembrolizumab and SD-101 groups were 4.66 cm (range 2–12), 5.09 (range 2.7–8.7) and 4.69 (range 1.3–8), respectively. Baseline FTV in the control, pembrolizumab and SD-101 groups was 17.48 (1.31–64.33), 24.37 (2.35–67.98), and 23.19 (0.03–71.03), respectively. When comparing between treatment groups, the chemo-immunotherapy group had a higher percentage of patients with HR + disease (17 patients, 63%) compared to the control group (8 patients, 50%). Additionally, the control group had lower baseline FTV (17.48 cc) compared to either chemo-immunotherapy arm (24.37 and 23.19 cc for pembrolizumab and SD-101 groups, respectively).

### Lymph node changes by treatment arm and time point

**Fig. 1:**
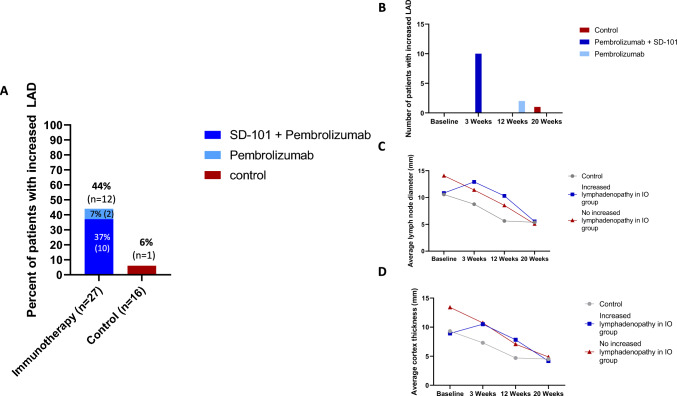
Lymph node change by treatment group. **a** Increased lymphadenopathy by treatment group. Percentage of patients with increased lymphadenopathy is depicted for each treatment group. Increased lymphadenopathy was defined as either an increase in the diameter of the largest abnormal ipsilateral lymph node or the appearance of new morphologically abnormal-appearing regional lymph nodes. Those undergoing immunotherapy with increased lymphadenopathy are depicted in blue while those undergoing control treatment with increased lymphadenopathy are depicted in red. **b** Increased lymphadenopathy by time point. Number of patients with increased lymphadenopathy is depicted for each treatment group by timepoint. Timepoints represent the first instance in which a patient developed new/increased adenopathy. MRI at each time point was compared to baseline imaging. Increased lymphadenopathy was defined as either an increase in the diameter of the largest abnormal ipsilateral lymph node or the appearance of new morphologically abnormal-appearing regional lymph nodes. Those undergoing immunotherapy with increased lymphadenopathy are depicted in blue while those undergoing control treatment with increased lymphadenopathy are depicted in red. **c** Average longest lymph node diameter grouped by changes to regional lymph nodes. MRI images at baseline, 3, 12 and 20 weeks were analyzed by a single radiologist that was blinded to treatment arm. Diameter size of the largest abnormal lymph node for each patient was noted with the average diameter depicted at each time point for those undergoing immunotherapy that experienced increased adenopathy (depicted in blue), those undergoing immunotherapy who did not experience increased regional adenopathy (depicted in red) and those undergoing control treatment (depicted in grey). **d** Average longest lymph node cortex thickness grouped by changes to regional lymph nodes. MRI images at baseline, 3, 12 and 20 weeks were analyzed by a single radiologist that was blinded to treatment arm. Cortex thickness of the largest abnormal lymph node for each patient was noted with the average thickness depicted at each time point for those undergoing immunotherapy that experienced increased adenopathy (depicted in blue), those undergoing immunotherapy who did not experience increased regional adenopathy (depicted in red) and those undergoing control treatment (depicted in grey). Abbreviations: LAD = lymphadenopathy

Of the 27 patients who received chemo-immunotherapy, 12 patients experienced either new lymphadenopathy or increase in diameter of the largest abnormal ipsilateral lymph node. This included 2 patients in the pembrolizumab group and 10 patients in the SD-101/pembrolizumab group (Table 2, Fig. 1a). 6 had an increase in number, 3 had an increase in diameter, and 3 had both. This change was statistically significant when compared to 1 patient in the control group who experienced increased diameter of regional lymph nodes (*p* = 0.0143; Fig. 1a–b, Table 2). The change in lymphadenopathy was primarily driven by changes in the SD-101/pembrolizumab arm compared to control (*p* = 0.002, Supplementary Table 1). Of note, 6 patients in the SD-101/pembrolizumab arm had enlargement of lymph nodes outside of ipsilateral axillary region, including the contralateral axillary nodes and ipsilateral internal mammary nodes (Supplementary Table 1). Of the 12 patients in the chemo-immunotherapy group who experienced larger or new lymphadenopathy, all changes occurred either at 3 or 12 weeks with subsequent decrease by week 20 (See Table 2, Fig. 1b). 11 out of these 12 patients were also found to be pathologically lymph node negative at the time of surgery (Table 4). The one patient in the control group who experienced increased lymphadenopathy developed this at 20 weeks (see Table 2, Fig. 1b) and was found to be node positive at surgery. Mean lymph node diameter of the largest abnormal lymph node and mean lymph node cortex size for all patients is depicted in Fig. 1c and d and Table 3. For those patients with increased lymphadenopathy, baseline diameter of the largest abnormal ipsilateral lymph node was 10.85 mm with an increase to 12.92 mm at 3 weeks and subsequent decrease (Table 3, Fig. 1c). Similarly, mean cortex thickness of the largest abnormal ipsilateral lymph node in the group with increased lymphadenopathy was 8.92 mm with an increase to 10.50mm at 3 weeks and subsequent decrease (Table 3, Fig. 1d). Average lymph node diameter and cortex thickness of the largest abnormal ipsilateral node is depicted by treatment arm in Table 3 and Supplementary Fig. 1.

**Table 2:** Lymph node changes

	Control	Pembrolizumab	SD-101 + pembrolizumab	Immunotherapy groups combined
*Number of patients with increase in lymph node diameter OR new ipsilateral axillary lymph nodes*				
Any time point	1	2	10	12
Baseline to 3 weeks	0	0	10	10
Baseline to 12 weeks	0	2	2	4
Baseline to 20 weeks	1	0	0	0
*Number of patients with increase in lymph node size*				
Any time point	0	0	6	6
Baseline to 3 weeks	0	0	6	6
Baseline to 12 weeks	0	0	5	5
Baseline to 20 weeks	0	0	1	1
*Number of patients developing NEW abnormal appearance in contralateral or internal mammary lymph nodes*				
Any time point	0	0	6	6
Baseline to 3 weeks	0	0	5	5
Baseline to 12 weeks	0	0	6	6
Baseline to 20 weeks	0	0	2	2

**Table 3:** Average lymph node size

*Average lymph node size of largest abnormal ipsilateral node grouped by change in lymph node*			
	Increase in lymph node size/number in immunotherapy group (*n* = 12)	No increase in lymph node size/number in immunotherapy group (*n* = 15)	Control group (*n* = 16)
*Average lymph node diameter in mm (range)*			
Baseline	10.85 (3–22)	14.07 (3–24)	10.56 (3–28)
3 weeks	12.92 (8–22)	11.40 (3–20)	8.75 (3–15)
12 weeks	10.31 (3–15)	8.53 (3–18)	5.63 (3–12)
20 weeks	5.54 (3–15)	5.07 (3–13)	5.38 (3–9)
*Average lymph node cortex size in mm (range)*			
Baseline	8.92 (3–22)	13.40 (3–24)	9.31 (3–28)
3 weeks	10.50 (5–22)	10.73 (3–20)	7.31 (3–15)
12 weeks	7.83 (23–14)	7.07 (3–18)	4.69 (3–12)
20 weeks	4.17 (3–11)	4.87 (3–13)	4.50 (3–11)

Average lymph node size of largest abnormal ipsilateral node grouped by treatment arm				
	Control	Pembrolizumab	SD-101 + pembrolizumab	Immunotherapy groups combined
Average lymph node diameter in mm (range)				
Baseline	10.56 (3–28)	11.91 (3–24)	13.00 (3–23)	12.56 (3–24)
3 weeks	8.75 (3–15)	10.18 (3–20)	13.38 (8–22)	12.07 (3–22)
12 weeks	5.63 (3–12)	7.64 (3–18)	10.50 (3–16)	9.33 (3–16)
20 weeks	5.38 (3–9)	4.64 (3–13)	5.25 (3–15)	5.00 (3–15)
*Average lymph node cortex size in mm (range)*				
Baseline	9.31 (3–28)	10.73 (3–24)	11.88 (3–23)	11.41 (3–24)
3 weeks	7.31 (3–15)	8.91 (3–20)	11.81 (6–22)	10.63 (3–22)
12 weeks	4.69 (3–12)	5.64 (3–18)	8.63 (3–16)	7.41 (3–18)
20 weeks	4.50 (3–11)	4.45 (3–13)	4.63 (3–11)	4.56 (3–13)

### Lymph node change and clinicopathologic characteristics

**Fig. 2:**
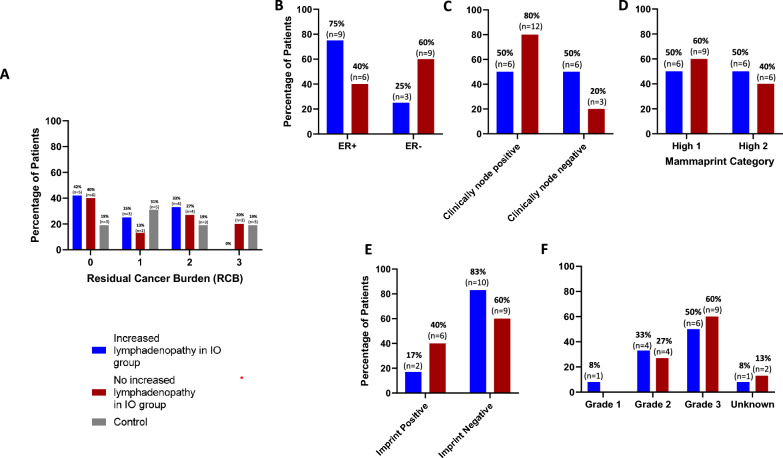
Lymph node changes and clinicopathologic characteristics. **a** Residual cancer burden by treatment group. Residual cancer burden (RCB) was calculated after surgery using previously established methods as described in manuscript. Increased lymphadenopathy was defined as either an increase in the diameter of the largest abnormal ipsilateral lymph node or the appearance of new morphologically abnormal-appearing regional lymph nodes. Patients undergoing immunotherapy with increased lymphadenopathy are depicted in blue while those without increased lymphadenopathy are depicted in red. RCB was also calculated in patients undergoing control treatment with chemotherapy alone, depicted in grey. **b** Estrogen receptor status and lymphadenopathy in patients undergoing immunotherapy. Changes in lymphadenopathy were assessed in patients with estrogen receptor positive disease and estrogen receptor negative disease for patients undergoing immunotherapy treatment. Patients undergoing immunotherapy with increased lymphadenopathy are depicted in blue while those without increased lymphadenopathy are depicted in red. **c** Clinical node status and lymphadenopathy in patients undergoing immunotherapy. Changes in lymphadenopathy were assessed for patients deemed to have clinically node positive and negative disease prior to treatment initiation. Patients were defined as clinically node positive if they had a lymph node biopsy that was positive for malignancy or if explicitly stated by the treating physician. **d** Mammaprint and lymphadenopathy in patients undergoing immunotherapy. Changes in lymphadenopathy were assessed for patients based on Mammaprint score. All patients in ISPY2 trial were required to have Mammaprint high-risk disease in order to receive immunotherapy. Mammaprint High 1 was defined as any score between 0 and − 0.56 while High 2 is defined as a score less than − 0.56. e Imprint status and lymphadenopathy in patients undergoing immunotherapy. Changes in lymphadenopathy were assessed for patients with positive and negative Imprint assay. Imprint is a multigenomic panel developed through analysis of patients on the ISPY trial designed to predict those patients most likely to respond to immune checkpoint inhibition. f Grade and lymphadenopathy in patients undergoing immunotherapy. Changes in lymphadenopathy were assessed for patients based on grade of tumor at time of diagnosis. Abbreviations: IO = immunotherapy; ER +  = estrogen receptor positive; ER− = estrogen receptor negative

In patients who received chemo-immunotherapy, 8 of 12 patients (66.7%) with increased lymphadenopathy achieved RCB of 0 or 1, compared to 8 of 15 (53.3%) patients without increased lymphadenopathy (*p* = 0.696, see Fig. 2, Table 4). Four (33.3%) patients with increased lymphadenopathy had RCB2 or 3, compared to 7 (46.7%) patients without increased lymphadenopathy. When evaluating the SD101 and pembrolizumab arm alone, 7 of 10 (70%) with increased lymphadenopathy achieved RCB of 0 or 1, compared to 2 of 6 (33.3%) without increased lymphadenopathy (*p* = 0.302, Supplementary Table 1). In patients with TNBC, 3 (25%) had increased lymphadenopathy and in patients with HR + /HER2 + breast cancer, 9 (75%) had increased lymphadenopathy. Increased lymphadenopathy was observed in 6 (33%) patients with clinically node positive disease and 6 (67%) patients with clinically node negative disease. In patients with increased lymphadenopathy, there was an even distribution of MammaPrint(30) H1 and H2 (6 patients, 50%) and in the patients without increased lymphadenopathy there were 9 (60%) patients with H1 and 6 patients (40%) with H2. Two patients (16.7%) were ImPrint positive in the group with increased lymphadenopathy compared to 6 (40%) in the patients without increased lymphadenopathy. Finally, the majority of patients in both groups had grade 3 tumors, with 6 (50%) in the increased lymphadenopathy group and 9 (60%) in the group with no change or decreased lymphadenopathy.

**Table 4:** Lymph node change and clinical characteristics/outcomes

	Increase in lymph node size/number in immunotherapy group (*n* = 12)	No increase in lymph node size/number in immunotherapy group (*n* = 15)	Control group (*n* = 16)
RCB			
RCB 0 or 1	8 (66.7%)	8 (53.3%)	9 (56.3%)
0	5 (41.7%)	6 (40.0%)	4 (25.0%)
1	3 (25.0%)	2 (13.3%)	5 (31.3%)
RCB 2 or 3	4 (33.3%)	7 (46.7%)	7 (43.8%)
2	4 (33.3%)	4 (26.7%)	4 (25.0%)
3	0 (0%)	3 (20.0%)	3 (18.8%)
Subtype			
TNBC	3 (25.0%)	9 (60.0%)	8 (50.0%)
HR + /HER2−	9 (75.0%)	6 (40.0%)	8 (50.0%)
Clinical node status at baseline			
Positive	6 (50.0%)	12 (80.0%)	9 (56.3%)
Negative	6 (50.0%)	3 (20.0%)	6 (37.5%)
Pathologic node status at surgery			
Positive	1 (8.3%)	7 (46.7%)	7 (43.8%)
Negative	11 (91.7%)	8 (53.3%)	9 (56.3%)
Mammaprint			
High 1	6 (50.0%)	9 (60.0%)	8 (50.0%)
High 2	6 (50.0%)	6 (40.0%)	8 (50.0%)
ImPrint +	2 (16.7%)	6 (40.0%)	3 (18.8%)
Grade			
1	1 (8.3%)	0 (0%)	0 (0%)
2	4 (33.3%)	4 (26.7%)	3 (18.8%)
3	6 (50.0%)	9 (60.0%)	12 (75.0%)
Unknown	1 (8.3%)	2 (13.3%)	1 (6.3%)

*RCB* residual cancer burden; *HR* + hormone receptor positive; *HER2 −  *negative for HER2 amplification; *TNBC* triple negative breast cancer

### Lymph node change and immune-related toxicity

**Fig. 3:**
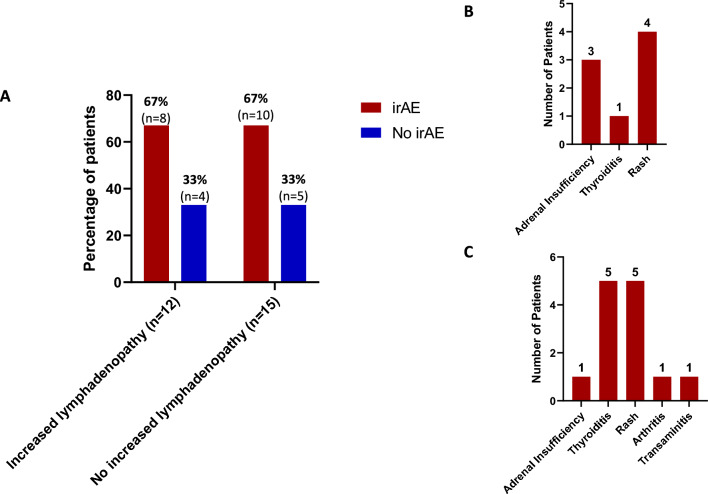
Immune-related adverse events in patients undergoing chemo-immunotherapy. **a** Lymph node changes in patients with and without immune-related adverse events. Incidence of immune-related adverse events was noted for patients undergoing immunotherapy with increased lymphadenopathy compared to those without increased lymphadenopathy. Increased lymphadenopathy was defined as either an increase in the diameter of the largest abnormal ipsilateral lymph node or the appearance of new morphologically abnormal-appearing regional lymph nodes. Patients who developed immune-related adverse events are depicted in red while those who did not are depicted in blue. **b** Types of immune-related adverse events in those with increased lymphadenopathy. Specific types of immune-related adverse events are depicted for patients undergoing immunotherapy with increased lymphadenopathy. **c** Types of immune-related adverse events in those without increased lymphadenopathy. Specific types of immune-related adverse events are depicted for patients undergoing immunotherapy without increased lymphadenopathy

We evaluated immune toxicities of patients undergoing ICI with pembrolizumab alone or pembrolizumab with SD-101 as outlined in Fig. 3. Of patients who experienced increased lymphadenopathy, 8 (66.7%) also experienced an immune-related adverse event (irAE) including adrenal insufficiency (*n* = 3), rash (*n* = 4), thyroiditis (*n* = 1). In patients undergoing ICI who did not experience a change in lymphadenopathy, 10 (66.7%) experienced an irAE including adrenal insufficiency (*n* = 1), rash (*n* = 5), thyroiditis (*n* = 5), transaminitis (*n* = 1) and arthritis (*n* = 1).

### Breast tumor size and volume

**Fig. 4:**
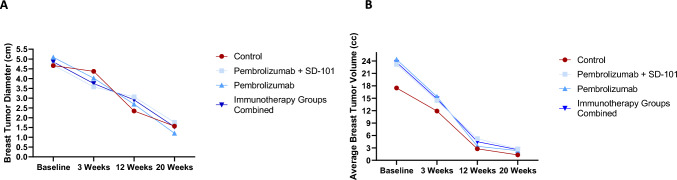
Changes in breast tumor size and volume by treatment group. **a** Changes in mean longest breast tumor diameter by treatment group. Breast tumor longest diameter size was assessed for each individual patient by MRI at baseline, 3, 12 and 20 weeks after start of therapy by a single radiologist who was blinded to treatment arm. The mean diameter is depicted by time point above. Patients receiving control treatment are depicted in red while patients receiving immunotherapy are depicted in blue. **b** Changes in mean functional breast tumor volume by treatment group. Breast tumor functional tumor volume was assessed by MRI using methods previously described in the manuscript at baseline, 3, 12 and 20 weeks after start of therapy. The mean functional tumor volume is depicted above with red indicating patients receiving control therapy and blue indicating patients receiving immunotherapy

Average longest breast tumor diameter and FTV are outlined in Table 5 and Fig. 4. In all three treatment groups, breast tumor diameter and FTV decreased at each time point. Baseline FTV and percent decrease in FTV over time were higher in patients who received chemo-immunotherapy compared to controls, including patients who had a concomitant increase in regional LNs.

**Table 5:** Breast tumor changes

	Control	Pembrolizumab	SD-101 + pembrolizumab	Immunotherapy groups combined
Average breast tumor diameter in cm (standard deviation)				
Baseline	4.66 (2.66)	5.09 (2.01)	4.69 (1.70)	4.85 (1.84)
3 weeks	4.37 (3.15)	4.02 (2.02)	3.58 (2.82)	3.75 (2.55)
12 weeks	2.34 (3.04)	2.69 (2.45)	3.06 (2.39)	2.91 (2.42)
20 weeks	1.57 (3.02)	1.21 (1.66)	1.77 (2.29)	1.54 (2.08)
Average change in breast tumor diameter in cm				
Baseline to 3 weeks	− 0.29	− 1.03	− 1.11	− 1.08
Baseline to 12 weeks	− 2.32	− 2.40	− 1.63	− 1.94
Baseline to 20 weeks	− 3.19	− 3.89	− 2.92	− 3.31
Average functional breast tumor volume in cc (standard deviation)				
Baseline	17.48 (18.72)	24.37 (18.71)	23.19 (20.19)	23.67 (19.61)
3 weeks	11.91 (14.99)	15.39 (19.16)	14.47 (9.15)	14.85 (14.21)
12 weeks	2.79 (4.18)	3.39 (5.67)	5.24 (7.54)	4.49 (6.90)
20 weeks	1.31 (2.77)	2.32 (4.45)	2.73 (4.34)	2.56 (4.44)
Change in functional breast tumor volume				
Baseline to 3 weeks	− 5.56	− 8.98	− 8.72	− 8.82
Baseline to 12 weeks	− 15.75	− 20.78	− 19.67	− 19.18
Baseline to 20 weeks	− 16.41	− 21.97	− 22.22	− 21.11

*Pre-op* pre-operative

## Discussion

With the increasing use of ICI in the treatment of breast cancer, early signs of treatment benefit as well as predictors of immune-related toxicity are needed in order to select patients for whom the benefits of immunotherapy outweigh the risks. Early radiologic markers of response represent one possible predictive tool, particularly with the use of serial breast MRIs, which are already commonly used in the setting of neoadjuvant therapy [9, 11, 13]. Prior serial breast MRI evaluation has focused on changes with neoadjuvant chemotherapy alone, with decreasing tumor size and FTV predictive of response [9, 13, 16, 17, 19]. However, in the case of treatment with ICI, the phenomenon of “pseudo-progression” has been described in a variety of tumor types in which initial increase in tumor size and volume, thought to be due to immune cell infiltration, are followed by subsequent treatment response. This phenomenon suggests that there may be unique patterns of imaging changes associated with chemo-immunotherapy as opposed to chemotherapy alone [26, 33, 34]. In addition, very little is known regarding regional lymph node changes on imaging in response to neoadjuvant chemo-immunotherapy and whether increased adenopathy may be a sign of disease progression or of immune activation. In this study, we aimed to evaluate changes in regional lymphadenopathy in patients with stage 2–3 breast cancer undergoing neoadjuvant chemo-immunotherapy compared to those receiving chemotherapy alone. To our knowledge, this is the first study to evaluate lymph node changes in patients with early breast cancer undergoing neoadjuvant chemo-immunotherapy, and to explore potential relationships with surgical pathology and other patient characteristics.

We found that patients undergoing chemo-immunotherapy, primarily with paclitaxel, pembrolizumab, and SD-101were more likely to develop increased lymphadenopathy compared to patients undergoing chemotherapy alone (44 vs 6.6%, *p* = 0.0143, Fig. 1, Table 2). Lymphadenopathy increased within the first 12 weeks of treatment prior to decreasing on subsequent therapy and occurred in a larger percentage of patients undergoing SD-101 with pembrolizumab compared to pembrolizumab alone. Of note, we found that SD-101 injections results in both ipsilateral and contralateral lymphadenopathy and may be informative for MRI monitoring of other trials utilizing localized therapies combined with systemic therapies. Similar findings have been reported in patients with non-small cell lung cancer undergoing neoadjuvant ICI who developed morphologically abnormal lymph nodes after treatment that upon biopsy were devoid of cancer cells but did show development of granulomatous inflammation [35]. Interestingly, despite early changes in lymphadenopathy, we note a persistent decrease in breast tumor size at all time points in both chemo-immunotherapy and control groups (Fig. 4, Table 5). These results suggest that patients undergoing chemo-immunotherapy may be more likely to experience early lymph node changes that are independent of tumor response to treatment. One reason for this may be that chemo-immunotherapy activates immune cells residing in regional lymph nodes against tumor cells in the breast. Prior preclinical studies in mouse models have demonstrated a persistent peripheral immune activation in regional lymph nodes being associated with ongoing tumor response to chemo-immunotherapy [36]. Evaluation of patients with head and neck squamous cell cancer has previously shown an increase in CD8 + T cell activity in uninvolved regional lymph nodes in response to immune checkpoint inhibitor treatment [37]. Thus, it may be that an early increase in lymphadenopathy seen on imaging may be reflective of increased peripheral immune activation against tumor.

The overall young age of patients in this cohort (median age 45) which may lead to heightened immune activation, and thus more pronounced lymph node imaging changes on MRI, compared to older patients who typically experience immunosenescence with age [38]. Furthermore, all patients in this cohort had early-stage disease. It is unclear if increasing lymphadenopathy with chemo-immunotherapy would be seen in patients with metastatic disease, particularly for those who are heavily pre-treated, as prior treatment may impair or alter the immune response to ICI. The majority of cases of increased lymphadenopathy were seen in patients who received intra-tumoral SD-101 in combination with ICI which is proof of concept that intra-tumor injection can potentially increase tumor immunogenicity, a key strategy for overcoming immunotherapy resistance.. While fewer patients receiving chemotherapy and pembrolizumab alone had increased regional lymphadenopathy, it is important to note that two patients in the pembrolizumab arm (without SD101) experienced new lymph nodes at 12 weeks and were subsequently node negative with decreased breast tumor size at the time of surgery. As immune checkpoint inhibitors are combined with other investigational agents in the future, it is important to understand the imaging patterns of various combinations in order to optimize clinical trial design and inform clinical practice.

When looking at RCB after neoadjuvant chemo-immunotherapy, there was no significant difference between rates of RCB 0 or 1 in patients with increasing lymphadenopathy compared to those without, though the absolute percentage of patients achieving RCB 0 or 1 was higher in those with increased lymphadenopathy (66.7 vs 53.3%, p = 0.696, Fig. 2, Table 4). There were also similar rates of RCB 2 or 3 in patients with increasing lymphadenopathy compared to those without (33.3 vs 46.7%). As noted above, primary tumor parameters improved equally in both cohorts, suggesting the increased lymphadenopathy does not represent new metastatic disease/disease progression and, if reproduced in larger cohorts, does not warrant sampling mid-therapy. This is supported by the fact that, of the 12 patients who experienced increased lymphadenopathy within the first 12 weeks, 11 (92%) had subsequent decrease in lymphadenopathy over the entire 20 weeks of neoadjuvant therapy with the remaining patient experiencing stable lymphadenopathy. In addition, 11 (92%) of these 12 patients were found to have pathologically negative lymph nodes at the time of surgery. This is despite 6 of these patients (50%) having biopsy-proven positive nodes prior to starting chemo-immunotherapy. While these trends were not significant, it is possible that this is largely due to the small sample size in our study and further studies in larger cohorts are needed to explore the association of imaging lymphadenopathy and treatment response. We did note that patients undergoing chemo-immunotherapy achieved RCB 0 at a higher rate compared to control (41 vs 25%) which is consistent with larger phase III trials showing increased rates of pCR with the addition of ICI to chemotherapy [1, 4, 39].

Our results did not indicate a correlation between estrogen receptor status, clinical lymph node status, MammaPrint score, Imprint score or tumor grade in the development of increased lymphadenopathy. We also observed similar rates of irAE in patients with increased lymphadenopathy compared to those without (66.7% in both groups), suggesting that regional lymphadenopathy may not predict development of off-target immune activity in our small sample size. This may point to differential mechanisms of immune activation associated with regional lymphadenopathy compared to development of systemic adverse events. While at this time this finding is hypothesis-generating for future investigation, it suggests the possibility of distinct immune mechanisms of response as opposed to toxicity.

As previously mentioned, one major limitation of this study is the small sample size which may lead to bias or lack of generalizability. In addition, we note that most lymph node increases were observed in patients undergoing SD-101 in combination with pembrolizumab and chemotherapy, indicating that our results may not be generalizable to larger groups of patients who undergo ICI and chemotherapy alone. Finally, MRI may not be the optimal imaging modality to assess lymph node size, as ultrasound is often the method of choice for evaluating suspicious axillary nodes [40]. However, as MRI is the best modality to evaluate neoadjuvant treatment response, findings on this modality need to be understood.

In conclusion, we found that patients undergoing neoadjuvant chemo-immunotherapy with pembrolizumab with or without SD-101 were more likely to experience early increased lymphadenopathy on serial MRI within the first 12 weeks of treatment despite concomitant decreases in breast tumor size. This effect was driven by the SD-101/pembrolizumab arm. The findings suggest that increasing adenopathy in this clinical context is unlikely to represent disease progression and it may be reasonable to defer follow-up imaging in the neoadjuvant setting. We did not observe a correlation between residual cancer burden, clinical node status, MammaPrint high-risk score, Imprint score, tumor ER status or tumor grade with increased lymphadenopathy and patients experienced similar rates of irAE regardless of lymph node changes. Future studies are warranted to determine whether on-treatment increases in lymphadenopathy may be an early sign of treatment response to neoadjuvant chemo-immunotherapy.

## Supplementary Information

Below is the link to the electronic supplementary material.

Supplementary Figure 1. Changes to lymph node size by treatment group. Supplementary Figure 1A: Average longest lymph node diameter by treatment group. MRI images at baseline, 3 weeks, 12 weeks and 20 weeks were analyzed by a single radiologist that was blinded to treatment arm. Diameter size of the largest abnormal lymph node for each patient was noted with the average diameter size is depicted at each time point by treatment group. The red line represents average diameter size of the largest abnormal lymph node in patients receiving control treatment while blue lines depict those receiving immunotherapy. Supplementary Figure 1B: Average longest lymph node cortex thickness by treatment group. MRI images at baseline, 3 weeks, 12 weeks and 20 weeks were analyzed by a single radiologist that was blinded to treatment arm. Diameter size of the largest abnormal lymph node cortex for each patient was noted with the average cortex diameter size is depicted at each time point by treatment group. The red line represents average cortex diameter size of the largest abnormal lymph node in patients receiving control treatment while blue lines depict patients receiving immunotherapy.
Supplementary file1 (PDF 31 KB)

Supplementary file2 (DOCX 15 KB)

## Data Availability

The datasets generated during and/or analysed during the current study are not publicly available but are available from the corresponding author on reasonable request.

## References

[1] Schmid P, Cortes J, Pusztai L, McArthur H, Kümmel S, Bergh J et al (2020) Pembrolizumab for early triple-negative breast cancer. N Engl J Med 382(9):810–821 32101663 10.1056/NEJMoa1910549

[2] Cortes J, Rugo HS, Cescon DW, Im SA, Yusof MM, Gallardo C et al (2022) Pembrolizumab plus chemotherapy in advanced triple-negative breast cancer. N Engl J Med 387(3):217–226 35857659 10.1056/NEJMoa2202809

[3] Mayer EL, Ren Y, Wagle N, Mahtani R, Ma C, DeMichele A, et a (2023) Abstract GS3–06: GS3–06 Palbociclib after CDK4/6i and endocrine therapy (PACE): a randomized phase II study of fulvestrant, palbociclib, and avelumab for endocrine pre-treated ER+/HER2-metastatic breast cancer. Cancer Res 83(5_Supplement):GS3–06.

[4] Cardoso F, McArthur HL, Schmid P, Cortés J, Harbeck N, Telli ML et al (2023) LBA21 KEYNOTE-756: phase III study of neoadjuvant pembrolizumab (pembro) or placebo (pbo) + chemotherapy (chemo), followed by adjuvant pembro or pbo + endocrine therapy (ET) for early-stage high-risk ER+/HER2– breast cancer. Ann Oncol 34:S1260–S1261

[5] Sharpe AH, Pauken KE (2018) The diverse functions of the PD1 inhibitory pathway. Nat Rev Immunol 18(3):153–167 28990585 10.1038/nri.2017.108

[6] Martins F, Sofiya L, Sykiotis GP, Lamine F, Maillard M, Fraga M et al (2019) Adverse effects of immune-checkpoint inhibitors: epidemiology, management and surveillance. Nat Rev Clin Oncol 16(9):563–580 31092901 10.1038/s41571-019-0218-0

[7] Nunes Filho P, Albuquerque C, Pilon Capella M, Debiasi M (2023) Immune checkpoint inhibitors in breast cancer: a narrative review. Oncol Ther 11(2):171–183 36917399 10.1007/s40487-023-00224-9 PMC10260715

[8] Hylton N (2006) MR Imaging for assessment of breast cancer response to neoadjuvant chemotherapy. Magn Reson Imaging Clin N Am 14(3):383–389 17098179 10.1016/j.mric.2006.09.001

[9] Esserman L, Hylton N, Yassa L, Barclay J, Frankel S, Sickles E (1999) Utility of magnetic resonance imaging in the management of breast cancer: evidence for improved preoperative staging. J Clin Oncol Off J Am Soc Clin Oncol 17(1):110–119 10.1200/JCO.1999.17.1.110 10458224

[10] Van Goethem M, Schelfout K, Dijckmans L, Van Der Auwera JC, Weyler J, Verslegers I et al (2004) MR mammography in the pre-operative staging of breast cancer in patients with dense breast tissue: comparison with mammography and ultrasound. Eur Radiol 14(5):809–816 14615904 10.1007/s00330-003-2146-7

[11] Davis PL, Staiger MJ, Harris KB, Ganott MA, Klementaviciene J, McCarty KS et al (1996) Breast cancer measurements with magnetic resonance imaging, ultrasonography, and mammography. Breast Cancer Res Treat 37(1):1–9 8750522 10.1007/BF01806626

[12] Partridge SC, Gibbs JE, Lu Y, Esserman LJ, Sudilovsky D, Hylton NM (2002) Accuracy of MR imaging for revealing residual breast cancer in patients who have undergone neoadjuvant chemotherapy. AJR Am J Roentgenol 179(5):1193–1199 12388497 10.2214/ajr.179.5.1791193

[13] Fatayer H, Sharma N, Manuel D, Kim B, Keding A, Perren T et al (2016) Serial MRI scans help in assessing early response to neoadjuvant chemotherapy and tailoring breast cancer treatment. Eur J Surg Oncol J Eur Soc Surg Oncol Br Assoc Surg Oncol 42(7):965–972 10.1016/j.ejso.2016.03.019 27260848

[14] Li H, Yao L, Jin P, Hu L, Li X, Guo T et al (2018) MRI and PET/CT for evaluation of the pathological response to neoadjuvant chemotherapy in breast cancer: A systematic review and meta-analysis. The Breast 1(40):106–115 10.1016/j.breast.2018.04.018 29758503

[15] Yeh E, Slanetz P, Kopans DB, Rafferty E, Georgian-Smith D, Moy L et al (2005) Prospective comparison of mammography, sonography, and MRI in patients undergoing neoadjuvant chemotherapy for palpable breast cancer. AJR Am J Roentgenol 184(3):868–877 15728611 10.2214/ajr.184.3.01840868

[16] Partridge SC, Gibbs JE, Lu Y, Esserman LJ, Tripathy D, Wolverton DS et al (2005) MRI measurements of breast tumor volume predict response to neoadjuvant chemotherapy and recurrence-free survival. Am J Roentgenol 184(6):1774–1781 15908529 10.2214/ajr.184.6.01841774

[17] Musall BC, Abdelhafez AH, Adrada BE, Candelaria RP, Mohamed RMM, Boge M et al (2021) Functional tumor volume by fast dynamic contrast-enhanced MRI for predicting neoadjuvant systemic therapy response in triple-negative breast cancer. J Magn Reson Imaging 54(1):251–260 33586845 10.1002/jmri.27557 PMC11830147

[18] Hylton NM, Gatsonis CA, Rosen MA, Lehman CD, Newitt DC, Partridge SC et al (2016) Neoadjuvant chemotherapy for breast cancer: functional tumor volume by MR imaging predicts recurrence-free survival—results from the ACRIN 6657/CALGB 150007 I-SPY 1 TRIAL. Radiology 279(1):44–55 26624971 10.1148/radiol.2015150013 PMC4819899

[19] Jafri NF, Newitt DC, Kornak J, Esserman LJ, Joe BN, Hylton NM (2014) Optimized breast MRI functional tumor volume as a biomarker of recurrence-free survival following neoadjuvant chemotherapy. J Magn Reson Imaging 40(2):476–482 24347097 10.1002/jmri.24351 PMC4507716

[20] QuantumLeap Healthcare Collaborative. I-SPY Trial (Investigation of Serial Studies to Predict Your Therapeutic Response With Imaging And moLecular Analysis 2) [Internet]. clinicaltrials.gov; 2023 Jul [cited 2023 Aug 9]. Report No.: NCT01042379. Available from: https://clinicaltrials.gov/study/NCT01042379

[21] SD-101—Investigational Therapeutic Candidate [Internet]. 2022 [cited 2023 Aug 10]. Available from: https://trisaluslifesci.com/candidate-sd-101/

[22] Melisi D, Frizziero M, Tamburrino A, Zanotto M, Carbone C, Piro G et al (2014) Toll-like receptor 9 agonists for cancer therapy. Biomedicines 2(3):211–228 28548068 10.3390/biomedicines2030211 PMC5344222

[23] Humbert M, Guery L, Brighouse D, Lemeille S, Hugues S (2018) Intratumoral CpG-B promotes antitumoral neutrophil, cDC, and T-cell cooperation without reprograming tolerogenic pDC. Cancer Res 78(12):3280–3292 29588348 10.1158/0008-5472.CAN-17-2549

[24] Frank MJ, Reagan PM, Bartlett NL, Gordon LI, Friedberg JW, Czerwinski DK et al (2018) In situ vaccination with a TLR9 agonist and local low-dose radiation induces systemic responses in untreated indolent lymphoma. Cancer Discov 8(10):1258–1269 30154192 10.1158/2159-8290.CD-18-0743 PMC6171524

[25] Ribas A, Medina T, Kummar S, Amin A, Kalbasi A, Drabick JJ et al (2018) SD-101 in combination with pembrolizumab in advanced melanoma: results of a phase Ib. Multicenter Study Cancer Discov 8(10):1250–1257 30154193 10.1158/2159-8290.CD-18-0280 PMC6719557

[26] Chiou VL, Burotto M (2015) Pseudoprogression and immune-related response in solid tumors. J Clin Oncol 33(31):3541–3543 26261262 10.1200/JCO.2015.61.6870 PMC4622096

[27] Rugo Hope S, Olopade Olufunmilayo I, DeMichele A, Yau C, van ’t Veer Laura J, Buxton Meredith B et al (2016) Adaptive randomization of Veliparib–Carboplatin treatment in breast cancer. N Engl J Med 375(1):23–34. 27406347 10.1056/NEJMoa1513749 PMC5259561

[28] Park John W, Liu Minetta C, Yee D, Yau C, van ’t Veer Laura J, Symmans WF et al (2016) Adaptive randomization of Neratinib in early breast cancer. N Engl J Med 375(1):11–22. 27406346 10.1056/NEJMoa1513750 PMC5259558

[29] Symmans WF, Peintinger F, Hatzis C, Rajan R, Kuerer H, Valero V et al (2007) Measurement of residual breast cancer burden to predict survival after neoadjuvant chemotherapy. J Clin Oncol Off J Am Soc Clin Oncol 25(28):4414–4422 10.1200/JCO.2007.10.6823 17785706

[30] Cardoso F, van’t Veer LJ, Bogaerts J, Slaets L, Viale G, Delaloge S et al (2016) 70-Gene signature as an aid to treatment decisions in early-stage breast cancer. N Engl J Med 375(8):717–729. 27557300 10.1056/NEJMoa1602253

[31] Mittempergher L, Kuilman MM, Barcaru A, Nota B, Delahaye LJMJ, Audeh MW et al (2022) The ImPrint immune signature to identify patients with high-risk early breast cancer who may benefit from PD1 checkpoint inhibition in I-SPY2. J Clin Oncol 40(16_suppl):514–514.

[32] Brufsky AM, Kuilman M, Mukhtar R, Wolf DM, Yau C, O’Shaughnessy J et al (2023) Abstract PD9–08: ImPrint immune signature in 10,000 early-stage breast cancer patients from the real-world FLEX database. Cancer Res 83(5_Supplement):PD9–08.

[33] Hodi FS, Hwu WJ, Kefford R, Weber JS, Daud A, Hamid O et al (2016) Evaluation of immune-related response criteria and RECIST v1.1 in patients with advanced melanoma treated with Pembrolizumab. J Clin Oncol Off J Am Soc Clin Oncol 34(13):1510–1511. 10.1200/JCO.2015.64.0391 PMC5070547 26951310

[34] Di Giacomo AM, Danielli R, Guidoboni M, Calabrò L, Carlucci D, Miracco C et al (2009) Therapeutic efficacy of ipilimumab, an anti-CTLA-4 monoclonal antibody, in patients with metastatic melanoma unresponsive to prior systemic treatments: clinical and immunological evidence from three patient cases. Cancer Immunol Immunother CII 58(8):1297–1306 19139884 10.1007/s00262-008-0642-y PMC11030873

[35] Cascone T, Weissferdt A, Godoy MCB, William WN, Leung CH, Lin HY et al (2021) Nodal immune flare mimics nodal disease progression following neoadjuvant immune checkpoint inhibitors in non-small cell lung cancer. Nat Commun 12(1):5045 34413300 10.1038/s41467-021-25188-0 PMC8376947

[36] Spitzer MH, Carmi Y, Reticker-Flynn NE, Kwek SS, Madhireddy D, Martins MM et al (2017) Systemic immunity is required for effective cancer immunotherapy. Cell 168(3):487-502.e15 28111070 10.1016/j.cell.2016.12.022 PMC5312823

[37] Rahim MK, Okholm TLH, Jones KB, McCarthy EE, Liu CC, Yee JL et al (2023) Dynamic CD8+ T cell responses to cancer immunotherapy in human regional lymph nodes are disrupted in metastatic lymph nodes. Cell 186(6):1127-1143.e18 36931243 10.1016/j.cell.2023.02.021 PMC10348701

[38] Gruver AL, Hudson LL, Sempowski GD (2007) Immunosenescence of ageing. J Pathol 211(2):144–156 17200946 10.1002/path.2104 PMC1931833

[39] Loi S, McArthur HL, Harbeck N, Pusztai L, Delaloge S, Letrent K et al (2020) A phase III trial of nivolumab with neoadjuvant chemotherapy and adjuvant endocrine therapy in ER+/HER2- primary breast cancer: CheckMate 7FL. J Clin Oncol 38(15_suppl):TPS604–TPS604.

[40] Marino MA, Avendano D, Zapata P, Riedl CC, Pinker K (2020) Lymph node imaging in patients with primary breast cancer: concurrent diagnostic tools. Oncologist 25(2):e231–e242 32043792 10.1634/theoncologist.2019-0427 PMC7011661

