# Supplementary figures and images for: Regional lymph node changes on breast MRI in patients with early-stage breast cancer receiving neoadjuvant chemo-immunotherapy
Source: Breast Cancer Res Treat. 2024 Sep 21;209(1):147–59. doi: 10.1007/s10549-024-07481-w (PMC11785630; doi:10.1007/s10549-024-07481-w)

**A**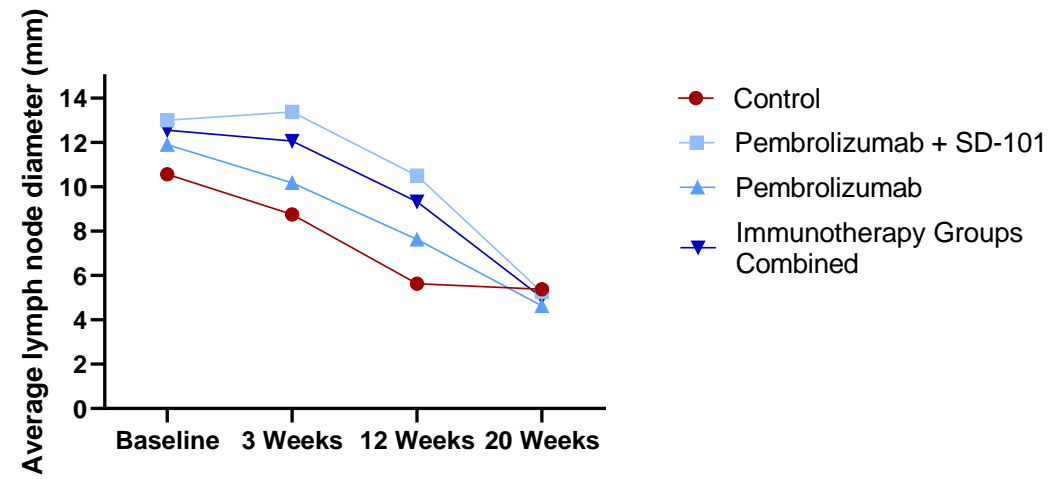**B**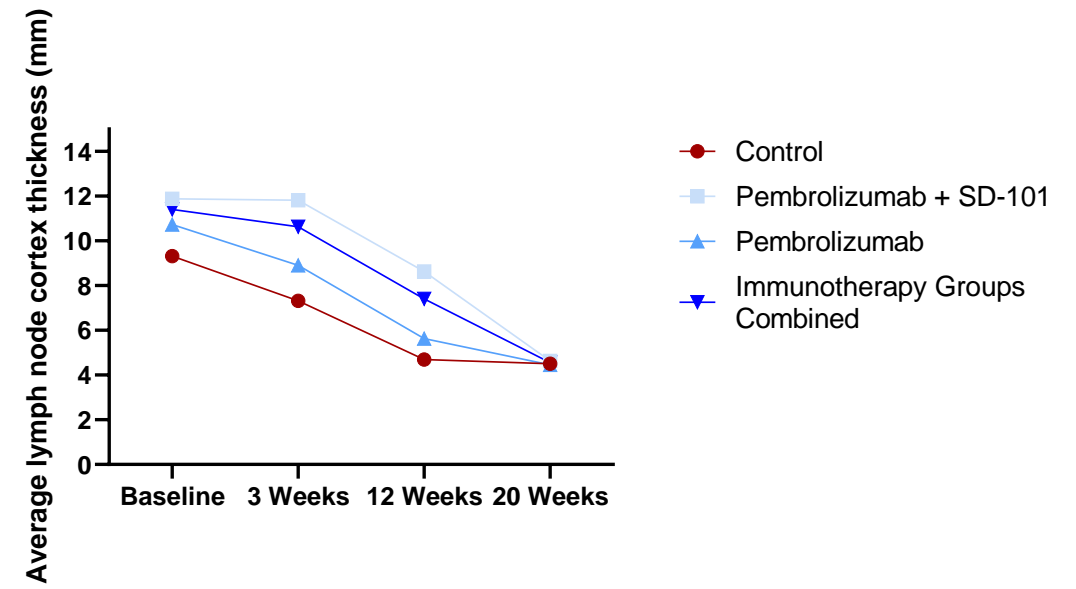

Supplement: Supplementary file 1 — Supplementary Figure 1. Changes to lymph node size by treatment group. Supplementary Figure 1A: Average longest lymph node diameter by treatment group. MRI images at baseline, 3 weeks, 12 weeks and 20 weeks were analyzed by a single radiologist that was blinded to treatment arm. Diameter size of the largest abnormal lymph node for each patient was noted with the average diameter size is depicted at each time point by treatment group. The red line represents average diameter size of the largest abnormal lymph node in patients receiving control treatment while blue lines depict those receiving immunotherapy. Supplementary Figure 1B: Average longest lymph node cortex thickness by treatment group. MRI images at baseline, 3 weeks, 12 weeks and 20 weeks were analyzed by a single radiologist that was blinded to treatment arm. Diameter size of the largest abnormal lymph node cortex for each patient was noted with the average cortex diameter size is depicted at each time point by treatment group. The red line represents average cortex diameter size of the largest abnormal lymph node in patients receiving control treatment while blue lines depict patients receiving immunotherapy. Supplementary file1 (PDF 31 KB) [file 10549_2024_7481_MOESM1_ESM.pdf]
