# Supplementary material for: Regional lymph node changes on breast MRI in patients with early-stage breast cancer receiving neoadjuvant chemo-immunotherapy
Source: Breast Cancer Res Treat. 2024 Sep 21;209(1):147–59. doi: 10.1007/s10549-024-07481-w (PMC11785630; doi:10.1007/s10549-024-07481-w)
Supplement: Supplementary file 2 — Supplementary file2 (DOCX 15 KB) [file 10549_2024_7481_MOESM2_ESM.docx]

SUPPLEMENTARY TABLE 1. LYMPH NODE CHANGES BY TREATMENT ARM IN SD-101 VS CONTROL

|  | CONTROL | | SD-101+PEMBROLIZUMAB | |
| --- | --- | --- | --- | --- |
| Number of patients with increase in lymph node diameter OR new ipsilateral axillary lymph nodes | | | | |
| *Any time point* | 0 | | 10 | |
| *Baseline to 3 weeks* | 0 | | 10 | |
| *Baseline to 12 weeks* | 0 | | 2 | |
| *Baseline to 20 weeks* | 1 | | 0 | |
| Number of patients with increase in lymph node size | | | | |
| *Any time point* | 0 | | 6 | |
| *Baseline to 3 weeks* | 0 | | 6 | |
| *Baseline to 12 weeks* | 0 | | 5 | |
| *Baseline to 20 weeks* | 0 | | 1 | |
| Number of patients developing NEW abnormal appearance in contralateral or internal mammary lymph nodes | | | | |
| *Any time point* |  | | 6 | |
| *Baseline to 3 weeks* | 0 | | 5 | |
| *Baseline to 12 weeks* | 0 | | 6 | |
| *Baseline to 20 weeks* | 0 | | 2 | |
| Average lymph node diameter of largest abnormal ipsilateral lymph node in mm (range) | | | | |
| *Baseline* | 10.56 (3-28) | | 13.00 (3-23) | |
| *3 weeks* | 8.75 (3-15) | | 13.38 (8-22) | |
| *12 weeks* | 5.63 (3-12) | | 10.50 (3-16) | |
| *20 weeks* | 5.38 (3-9) | | 5.25 (3-15) | |
| Average lymph node cortex size of largest abnormal ipsilateral lymph node in mm (range) | | | | |
| *Baseline* | 9.31 (3-28) | | 11.88 (3-23) | |
| *3 weeks* | 7.31 (3-15) | | 11.81 (6-22) | |
| *12 weeks* | 4.69 (3-12) | | 8.63 (3-16) | |
| *20 weeks* | 4.50 (3-11) | | 4.63 (3-11) | |
| **Characteristics by lymph node change in SD-101 arm vs control** | | | | |
|  | CONTROL (n=16) | Increased LAD in SD-101 arm (n=10) | | No increase in lymph node size/number in SD-101 arm (n=6) |
| Average lymph node diameter of largest abnormal ipsilateral lymph node in mm (range) | | | | |
| *Baseline* | 10.56 (3-28) | 10.90 (3-22) | | 16.50 (8-23) |
| *3 weeks* | 8.75 (3-15) | 13.80 (9-22) | | 12.67 (8-18) |
| *12 weeks* | 5.63 (3-12) | 10.90 (3-15) | | 9.86 (6-16) |
| *20 weeks* | 5.38 (3-9) | 5.00 (3-15) | | 5.67 (3-9) |
| Average lymph node cortex size of largest abnormal ipsilateral lymph node in mm (range) | | | | |
| *Baseline* | 9.31 (3-28) | 9.30 (3-14) | | 16.17 (6-23) |
| *3 weeks* | 7.31 (3-15) | 11.50 (6-22) | | 12.33 (6-18) |
| *12 weeks* | 4.69 (3-12) | 8.40 (3-14) | | 9.00 (4-16) |
| *20 weeks* | 4.50 (3-11) | 4.30 (3-11) | | 5.17 (3-9) |
| RCB | | | | |
| RCB 0 or 1 | 9 (56.3%) | 7 (70.0%) | | 2 (33.3%) |
| *RCB 0* | 4 (25.0%) | 4 (40.0%) | | 1 (16.7%) |
| *RCB 1* | 5 (31.3%) | 3 (30.0%) | | 1 (16.7%) |
| RCB 2 or 3 | 7 (43.8%) | 3 (30.0%) | | 4 (66.7%) |
| *RCB 2* | 4 (25.0%) | 3 (30.0%) | | 3 (50.0%) |
| *RCB 3* | 3 (18.8%) | 0 (0.0%) | | 1 (16.7%) |
| Subtype |  |  | |  |
| *TNBC* | 8 (50.0%) | 3 (30%) | | 3 (50.0%) |
| *HR+/HER2-* | 8 (50.0%) | 7 (70%) | | 3 (50.0%) |
| Clinical node status at baseline | | | | |
| *Positive* | 9 (56.3%) | 4 (40%) | | 6 (100.0%) |
| *Negative* | 6 (37.5%) | 6 (60%) | | 0 (0.0%) |
| Pathologic node status at surgery | | | | |
| *Positive* | 7 (43.8%) | 1 (10%) | | 4 (66.7%) |
| *Negative* | 9 (56.3%) | 9 (90%) | | 2 (33.3%) |
| Mammaprint |  |  | |  |
| *High 1* | 8 (50.0%) | 5 (50%) | | 4 (66.7%) |
| *High 2* | 8 (50.0%) | 5 (50%) | | 2 (33.3%) |
| ImPrint + | 3 (18.8%) | 1 (10%) | | 2 (33.3%) |
| Grade |  |  | |  |
| *1* | 0 (0%) | 1 (10%) | | 0 (0.0%) |
| *2* | 3 (18.8%) | 3 (30%) | | 2 (33.3%) |
| *3* | 12 (75.0%) | 5 (50%) | | 4 (66.7%) |
| *Unknown* | 1 (6.3%) | 1 (10%) | | 0 (0.0%) |

**Abbreviations:** RCB=residual cancer burden; HR+=hormone receptor positive; HER2-= negative for HER2 amplification; TNBC=triple negative breast cancer
